# Phylogeography of *Sarmarutilus rubilio* (Cypriniformes: Leuciscidae): Complex Genetic Structure, Clues to a New Cryptic Species and Further Insights into Roaches Phylogeny

**DOI:** 10.3390/genes13061071

**Published:** 2022-06-15

**Authors:** Gerardo Petrosino, Lorenzo Tancioni, Martina Turani, Arnold Rakaj, Luca Ciuffardi, Anna Rita Rossi

**Affiliations:** 1 Department of Biology and Biotechnology “C. Darwin”, Sapienza University of Rome, 00161 Rome, Italy; turanimartina@gmail.com (M.T.); annarita.rossi@uniroma1.it (A.R.R.); 2 Department of Biology, University of Rome “Tor Vergata”, 00133 Rome, Italy; tancioni@uniroma2.it (L.T.); arnoldrakaj@gmail.com (A.R.); 3 Center for BioNatural Studies srl, 16132 Genova, Italy; info@lucaciuffardi.it

**Keywords:** Italian ichthyogeographic districts, Albanian lakes, *Rutilus*, *Leucos*, freshwater fish, Cyfun P, cytochrome oxidase I, control region, vicariance events, secondary contacts, conservation genetics

## Abstract

Italy hosts a large number of endemic freshwater fish species due to complex geological events which promoted genetic differentiation and allopatric speciation. Among them, the South European roach *Sarmarutilus rubilio* inhabits various freshwater environments in three different ichthyogeographic districts. We investigated the genetic diversity of *S. rubilio* using two different mitochondrial markers (COI and CR), aiming to define its relationship with other similar taxa from the Balkan area and, from a phylogeographic perspective, test the effects of past hydrogeological dynamics of Italian river basins on its genetic structure and demographic history. Our analysis highlighted a marked genetic divergence between *S. rubilio* and all other roach species and, among Italian samples, revealed the existence of three deeply divergent geographic haplogroups, named A, B and C. Haplogroup C likely corresponds to a new putative cryptic species and is located at the northern border of the South European roach range; haplogroup B is restricted to Southern Italy; and haplogroup A is widespread across the entire range and in some sites it is in co-occurrence with C or B. Their origin is probably related to the tectonic uplifting of the Apuan Alps in the north and of the Colli Albani Volcano in the south during the Pleistocene, which promoted isolation and vicariance followed by secondary contacts.

## 1. Introduction

In recent decades, the integration of molecular approaches when investigating the diversity and distribution of animal species [1] has provided new insights into the taxonomy and evolution of freshwater fish, facilitating phylogenetic reconstruction [2,3], solving taxonomical controversies [4,5,6], identifying cryptic species [7,8,9], detecting the introduction of allochthonous species [10,11] and clarifying the conservation status of threatened species [12]. In addition, a strong relationship emerged between the genetic diversity of freshwater fishes, geography and past hydrogeological events [13,14,15].

The Mediterranean area is considered a biodiversity hotspot [16] and hosts 25% of the strictly freshwater fish species registered in the Palearctic region [17] due to its complex paleogeographic and paleoclimatic history. Indeed, tectonic movement in the Miocene and sea-level variations which occurred during the Messinian Salinity Crisis and Pleistocene glacial/interglacial periods, promoted genetic diversification and allopatric speciation across Mediterranean peninsulas, as these territories were alternatively linked and isolated from each other, and acted as glacial refugia during the Pleistocene [2,18]. Afterwards, mountainous ridges (e.g., Alps, Pyrenees) isolated native freshwater fishes from the post-glacial expansion of Eastern-European species [19], thus explaining the presence of the huge number of endemic species in this region (526 nominal and 490 confirmed [20]).

**Figure 1:**
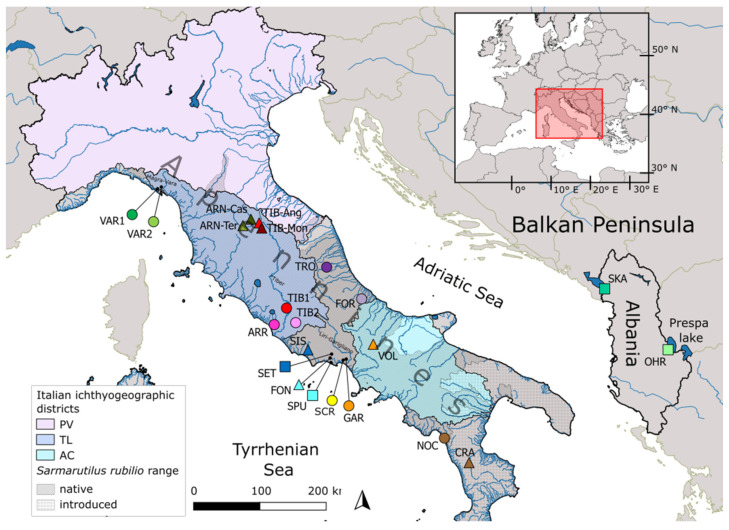
Geographic location of the study area (black and red rectangles, top right), along with *S. rubilio* distribution according to IUCN (https://www.iucnredlist.org/species/19786/9014268, accessed on 11 April 2022), and sampling sites: circle indicates rivers and square lakes. For abbreviations refer to Table 1. Triangles indicate sites corresponding to sequences retrieved from the Genbank database and included in the COI phylogenetic reconstruction. Names of major basins discussed in the paper are also indicated.

In the Italian peninsula, three different ichthyogeographic districts are currently recognized, named Padany-Venetian (PV), Tuscany-Latium (TL), and Apulia-Campania (AC) (see Figure 1), whose borders are still not clearly defined [21]. These districts were shaped by river confluence and isolation mechanisms during the Pleistocene and host different fish species assemblages [22], corresponding to 61 native species, 27 of which are endemic or sub-endemic [23]. Most of them are threatened by invasive species, which are equal to the number of native ones [23], and by other anthropogenic impacts such as habitat degradation, water pollution and abstraction [24]. Among the existing Italian native species, the Leuciscidae family (previously included in the Cyprinidae, see [25]), is the most represented group with 14 species (23% of native ones). Despite the use of molecular markers providing useful information about these species in the last decade [26,27,28], genetic data are still required for many of them for effective management, and conservation action plans are still lacking [29].

The South European roach *Sarmarutilus rubilio* (Bonaparte, 1837)—a unique species of the genus—is a leuscid previously known as *Rutilus rubilio*. Nomenclature change of this taxon was driven by the identification of diagnostic morphological differences scarcely appreciable with the naked eye and by separate clustering when compared to any other genetic lineage included in the former *Rutilus* genus [30]. It derived its name from the Sarmatic Sea, or Lago Mare, an ancient central European inner freshwater sea where it likely originated in the Middle Miocene, between 5 [30] and 9.7 [2] million years ago (Mya) when most of the Italian Peninsula was still under sea level. The South European roach has a broad ecological niche. It is eurytopic, moderately rheophilic and thermophilic, mainly living in hilly and lowland stretches of streams and rivers, and more rarely in lentic environments. It is distributed from North-Central to Southern Italy, with most of the population localized in the basins of the Tyrrhenian slope (from River Magra north to River Sele south) and some in the Adriatic basin (from River Tronto in the north to River Trigno in the south) according to [30] (see Figure 1 below); thus, its range includes the three different Italian ichthyogeographic districts, although it is mainly found in the TL district. In addition, *S. rubilio* was also translocated in many basins of the southern part of the Italian Peninsula and Sicily—even in Tunisia [31] and Turkey [32]—and the border between the native and introduced ranges still remains unclear. Presently, the number and size of its populations is declining and its range decreased by over 30% in 10 years [33], mainly due to the introduction of allochthonous species, such as the morphologically and ecologically similar roaches *Leucos aula* and *Rutilus rutilus* [34]; thus, even though the species is still classified in the Near-Threatened IUCN risk assessment category, it is likely going to be qualified as Vulnerable in the near future [33] and is listed in Annex II of the European Union Habitats Directive [35]. The presence of the species (or subspecies) was also reported in Albanian lakes [36] as external meristic traits between Italian *S. rubilio* and Albanian roaches overlap; therefore, debates over the taxonomical status of Albanian roach are ongoing [20,30,37]. *Sarmarutilus rubilio* distribution could likely disguise a strong genetic structure, which is also suggested by the evidence that this species is listed among those Mediterranean freshwater fish taxa (about 98% barcoded) characterized by high intraspecific clusters divergence; therefore qualifying it as a potential candidate species, i.e., cryptic species [20] (Appendix S1). Starting with these assumptions, we investigated both the complete mitochondrial control region (CR) in almost two hundred specimens from the whole species range, and the barcoding portion of the cytochrome oxydase I (COI) in a subset of Italian and Albanian *Sarmarutilus*-like individuals. In addition, we analyzed two nuclear markers (Cyprinid formerly unknown nuclear Polymorphism, and first intron of the ribosomal protein S7) in a few Italian specimens showing a highly divergent COI profile. These analyses aimed to (1) assess the relationship between Italian *S. rubilio* specimens from Albania with a similar morphology and other roach species (all species previously included in the genus *Rutilus* and now split among *Leucos*, *Sarmarutilus* and *Rutilus*); (2) test the presence of geographically localized South European roach genetic clusters in Italy; (3) make inferences on how the hydrogeological evolution of Italian peninsula influenced *S. rubilio* lineage diversification. The results will be useful in constructing future conservation action plans for this species and will provide further data on the relationships between Italy’s hydrogeological history and the genetic structure of freshwater fishes, thus contributing to the debate aimed at clarifying and eventually rearranging the Italian ichthyogeographic districts’ borders, in relation to the divergent processes of other endemic species.

## 2. Materials and Methods

### 2.1. Sampling, Morphological Identification and DNA Extraction

Overall, 223 specimens were collected, with 208 *S. rubilio* individuals from 12 sites in the Italian peninsula, covering most of the known supposed native range, from the Liguria region at the northern border of species distribution to the southern one in Basilicata. In addition, 15 specimens showing *S. rubilio*-like morphology were retrieved from two Albanian lakes, where this species was also reported in recent decades (Figure 1; Table 1).

Fishes were sampled using electrofishing and anaesthetized with a 0.035% MS 222 (Tricaine Methanesulfonate) solution, in accordance with the relevant legislation (CEN EN 131 14011/2003-Water quality-Sampling of fish with electricity) and standards for species listed in Annex II of Habitats Directive [38], as authorized by Regional Directions responsible for Hunting and Fishing activities and by Directions of Protected areas (Prot. n.: Lazio G10101, 25 July 2019; Abruzzo DPD023/171, 12 April 2021; Marche 213, 13 April 2021; Liguria 5166–2021, 30 August 2021; Aurunci protected area 0002963.U, 01 October 2021). Specimens were morphologically identified according to descriptions in the literature [39]. The tips of pelvic fins were taken from each specimen and fixed in 96% ethanol to further perform genetic analyses. After tissue collection and recovery from the anaesthetic, the specimens were released into the wild. Some entire specimens for each sampling site were preserved in 70% ethanol and deposited in the Museum of Comparative Anatomy “Battista Grassi”, Department of Biology and Biotechnology “Charles Darwin”, Sapienza University of Rome (AC1687-98). Total genomic DNA extraction was carried out using the salting-out method [40].

### 2.2. PCR Amplification

A fragment of approximately 620 base pairs (bp) of the mitochondrial cytochrome c oxidase subunit 1 (COI), widely used for animal species barcoding [41], was amplified in a subset of 2 to 11 specimens for each site to validate the morphological taxonomic identification. For other specimens (from VAR sites), molecular identification using COI was not resolutive, so we amplified and sequenced two different non-coding nuclear markers which were successfully used for species identification in Leuciscidae fishes [2,4], with 156–218 base pairs (bp) fragments of Cyprinid formerly unknown nuclear Polymorphism (Cyfun P, [42]), and 313–851 bp fragments of the first intron of the ribosomal protein S7 (S7).

To investigate genetic diversity across *S. rubilio* populations, the entire mitochondrial control region (CR, 930 bp), a non-coding fragment involved in the initiation of the replication and transcription of the entire mtDNA, was amplified in all Italian samples.

PCR reactions for each marker were carried out in a total volume of 20 μL in a TC9610 Multigene OptiMax Thermal Cycler. Reagents, primers description and thermal cycling condition parameters for each marker are described in Table S1. Amplicons were purified and sequenced using an external service (Microsynth AG, Balgach, Switzerland, www.microsynth.ch, accessed on 11 April 2022).

The obtained sequences were then compared to those available for almost all Leuciscidae species in BOLD [43] and Genbank using the Basic Local Alignment Search Tool (BLAST) [44].

### 2.3. Data Analysis

Sequences for all markers were aligned using Clustal X 2.0 [45] and the polymorphic sites were checked manually.

Mitochondrial haplotypes for both COI and CR were identified using DnaSP v6 [46] and haplotype networks were built using the TCS algorithm [47] in Popart [48].

To investigate the phylogenetic relationships with closely related taxa, we used COI sequences and applied the Maximum Likelihood (ML) and Bayesian Inference (BI) methods. Our COI dataset was enriched with all *S. rubilio* sequences stored in GenBank (19 in total) and with 4 sequences from each *Rutilus* and *Leucos* available species, which were taken from different basins when possible (Table S2); however, “unverified” sequences were not included. *Squalius cephalus* (accession number NC031540) was used as an outgroup. An ML analysis was performed using W-IQ-TREE [49], with the substitution model TIM + F + G4 defined by ModelFinder [50] according to the minimum BIC score, and by performing an ultrafast bootstrap analysis [51] for 1000 iterations for statistical support. BI analyses were performed using MrBayes 3.2.7 [52] with the GTR + I substitution model, selected by the AIC (Akaike Information Criterion) and implemented in JModelTest v.2.1.10 [53]. The analysis was carried out in three heated and one cold Metropolis-coupled Markov chain Monte Carlo (MCMC) in two independent runs of 1 million generations each, with sampling performed every 1000 generations and 10% of the trees discarded as burn-in. The effective sample size (ESS) of the two runs was evaluated using Tracer v1.7.1 [54] and each parameter exceeded 500. All trees obtained in the ML and BI analyses were visualized using FigTree v1.4.4 (Andrew Rambaut, Institute of Evolutionary Biology, University of Edinburgh, Edinburgh, UK, http://tree.bio.ed.ac.uk/software/figtree/, accessed on 11 April 2022).

After phylogenetic reconstruction, all the analyses of genetic diversity, population structure and demography were performed based on CR sequences of *S. rubilio* from Italy. Arlequin 3.1 [55] was used to calculate standard molecular indices (Hd and π, respectively [56]), both for populations and haplogroups identified by the network, and to calculate the genetic differentiation between populations as pairwise ΦST (Kimura 2-parameters distance–K2P [57]). Significance was tested by performing 10,000 permutations and adjusting *p*-values for multiple testing with Bonferroni correction. Additionally, inter- and intra-haplogroup genetic distances (K2P) were calculated using MEGA 11 [58]. Haplotype richness was calculated using the PopGenReport package [59] in R software [60] by randomly sampling 13 individuals per population to account for different sample sizes.

Population differences were visualized by performing non-metric multidimensional scaling (NMDS) in PAST 3.26 [61] on the ΦST matrix. The NMDS analysis was also repeated considering the presence of more than one haplogroup for some populations (see results).

An AMOVA (Analysis of Molecular Variance) with Arlequin 3.1 was used to test the various hypothesis of population structure, which were as follows: (1) no groups, i.e., panmixia, or population subdivision according to (2) ichthyogeographic district (3 groups, PV, TL, AC attributing sites located in the undefined area according to recent literature [27]), (3) number of haplogroups (3 groups, different from the previous hypothesis), (4) NMDS output (7 groups, due to the presence of subgroups within haplogroups).

The divergence time between CR haplogroups was estimated using the BI method implemented in BEAST v.1.10.4 [62], and by setting the HKY+I + G substitution model selected using JModelTest and the Coalescent Constant Size as Tree Prior; and 3.84 and 8.48% per million years (My) divergence rates were used, i.e., the minimum and maximum rates estimated for CR in Leuciscidae species [63]. *Rutilus rutilus*, the only roach species for which the CR sequence was available, and *Squalius squalus* (GenBank Accession Numbers AP010775 and NC031540, respectively), were used as outgroups. MCMC was run for 10 million steps and sampled every 1000 steps, with 10% of trees discarded as burn-in. The EES and trees were evaluated as in the COI analysis.

The ancestral state of haplogroup distribution, dispersal and vicariance events were inferred using the statistical DIVA (S-DIVA) method [64] implemented in RASP v.3.1 software [65]. CR Bayesian tree topology was used to map ancestral distributions and the tips of the tree (CR haplotypes) were coded using the following five main Italian hydrogeographic areas, identified a priori according to geographic distances and barriers between them: (1) Magra-Vara basin at the species northern border (VAR sites); (2) central Adriatic slope (TRO and FOR); (3) central Tyrrhenian slope (TIB sites and ARR); (4) Fondi plain (SET, SPU); (5) south Tyrrhenian slope (SCR, GAR, NOC). Outgroup species distribution was labeled as “continental Europe”.

To make inferences about past demographic events in *S. rubilio*, the following three steps were conducted for each previously identified CR haplogroup: (1) Arlequin 3.1 for the mismatch analysis and to test the demographic expansion hypothesis, calculating Harpending’s raggedness index Hri [66] and the sum of squared deviations SSD [67], which were both assessed with a parametric bootstrap of 10,000 replicates; and the expansion parameter τ and effective population size θ_0_ and θ_1_ before and after the expansion were also calculated; (2) the same software was used to perform Tajima’s D [68] and Fu’s F [69] neutrality tests together with the R2 neutrality test [70] implemented in DnaSP v6. When the significative signature of demographic expansion was found, the time since expansion was calculated as t = τ/(2μ * generation time * k), where 2μ is the divergence rate and K is sequence length [71]; the generation time for *S. rubilio* is one year [34].

## 3. Results

### 3.1. Molecular Identification and Phylogeny

The analysis of COI (624 bp) sequences from a subset of 69 specimens revealed 21 COI haplotypes (GenBank accessions: OM974277-97). A total of 7 of them were identified among the 15 sequences from Albanian specimens (differing from each other for one-three site-specific mutational steps) and 14 haplotypes from the sequences corresponding to 54 Italian samples (Table 1 and Figure S1). All variable sites between haplotypes were synonymous mutations. Comparisons with GenBank sequences for the Albanian samples showed > 99% similarity to sequences labelled as *Rutilus albus*, *R. prespensis*, *R. ohridanus* and only ~93% with *S. rubilio*. Considering Italian specimens, the morphological attribution to *S. rubilio* was confirmed by COI sequences for 46 out of the 54 individuals (from 97.91 to 100% similarity). The remaining eight specimens, collected in VAR1 and VAR2 sites (HpC01 and HpC02), showed 95.01–95.97% similarity with *S. rubilio* and lower values than any other species present in GenBank (92.72–94.55% when compared with other *Rutilus*/*Leucos* species). The BOLD identification engine was also unable to match any records in the selected database for these VAR specimens.

**Figure 2:**
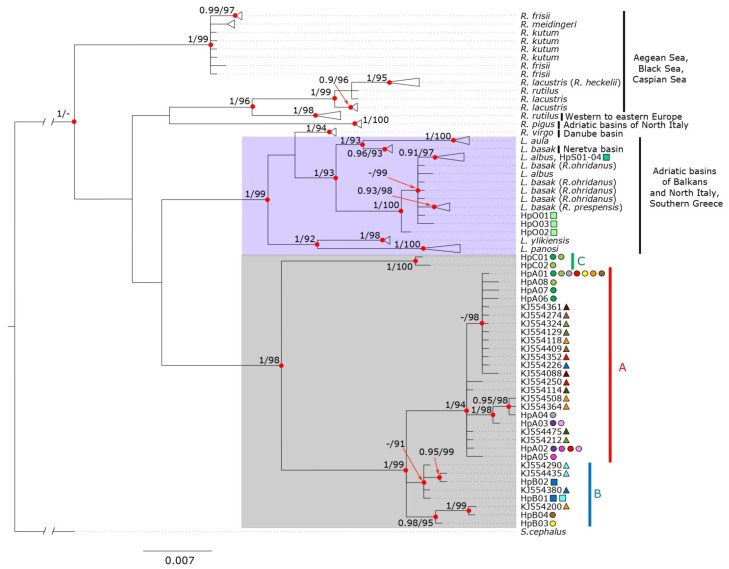
ML and BI phylogenetic tree based on mitochondrial sequences of COI including sequences of all the species originally included in the genus *Rutilus* (see Table S2 for Accession Number). Haplotypes obtained in this study are indicated and represented by circles (rivers) and squares (lakes); sequences retrieved from Genbank are indicated by triangles. Rectangles on the tree highlight currently recognized *Leucos* species (violet) and *Sarmarutilus* (grey); for the latter, three haplogroups are indicated. Posterior probabilities > 0.90 (BI) and bootstrap values > 90 (ML) at each node are shown.

A phylogenetic analysis including *Rutilus* and *Leucos* species showed the same tree topology for both ML and BI (Figure 2). All Albanian haplotypes from lake Skadar clustered with most *Leucos albus* specimens while those from lake Ohrid showed no clear assignment to a distinct group, but were still included with strong support in the *L. albus*/*R. ohridanus*/*R. prepspensis* cluster. Despite *R. ohridanus* (as well as *R. prespensis*) being currently considered a synonym of *Leucos basak* (see Discussion), our trees showed these putative taxa did not form a monophyletic group exclusive of *L. aula* or *L. albus*, and *L. basak* from the Neretva basin did not cluster with Albanian ones. Once it was ascertained that samples from Albania did not correspond to *S. rubilio*, they were excluded from further analysis.

All Italian sequences clustered in a well-supported monophyletic clade (hereafter *Sarmarutilus*); however, its relationships with *Leucos* and *Rutilus* clades were not resolved, as basal nodes in our tree were not supported. Within *Sarmarutilus*, different lineages were identified, with specific geographic distributions. The most differentiated one includes only two haplotypes from the sampling sites at the northern border of the species range (VAR, lineage C) and differs for 26–27 mutational steps from the most common haplotypes (HpA01 and HpB01) (Table S3a and Figure S1). The second (lineage A) includes the majority of the sequences and is widespread across the peninsula. The remaining sequences (group B) are exclusive of the southern Tyrrhenian slope and do not form a monophyletic cluster, although they are clearly grouped in the COI haplotype network (Figure S1), due to the low number of mutations compared to haplotypes of lineages A and C. From now on, we refer to haplogroups A, B and C. None of the previously deposited sequences clustered in haplogroup C.

Considering that COI haplogroup A (common *S. rubilio*) and C (unknown) coexist in the same VAR sites, we investigated whether (a) individuals from the two genetic lineages represent remnants of isolation and secondary contact events, (b) nuclear divergence among them still exist and/or (c) whether they hybridize. To test these hypotheses, nuclear markers were amplified in a subset of individuals from both mtDNA lineages (A and C), from VAR and sites in Central Italy. The nuclear S7 (GenBank accessions: OM966282-97) region did not show sufficient variability, and thus it did not provide an answer. On the contrary, the Cyfun P fragment (GenBank accessions: OM966266-81) was more variable and provided diagnostic mutations/indels that distinguished two groups of nuclear sequences among VAR specimens, one of which shared samples from Central Italy and the other was exclusive of VAR (Table S4). Sequences found in both VAR and Central Italy showed 97.02–100.00% similarity with the ones available in GenBank for *S. rubilio* (JQ286163-4, from Tiber river), while for those exclusive of VAR sites similarity values dropped to 90.30–92.20%. The genetic distance between our two groups of Cyfun P sequences (K2P, MEGA 11) was equal to approximately 4%, with VAR specific ones also distinguished by the deletion of nine base pairs. Of note, six out of seven individuals showing the typical Cyfun P VAR sequences belonged to the mitochondrial haplogroup C (Table S4). We re-examined the morphology of VAR specimens (pictures and vouchers) showing both mitochondrial and nuclear sequences belonging to lineage C, with no differences from *S. rubilio* observed (Figure S2) and meristic counts in the range of *S. rubilio* (number of branched rays of dorsal, anal and pelvic fins, scales on lateral line and number of pharyngeal teeth [30]).

### 3.2. Genetic Variability and Demographic History

**Figure 3:**
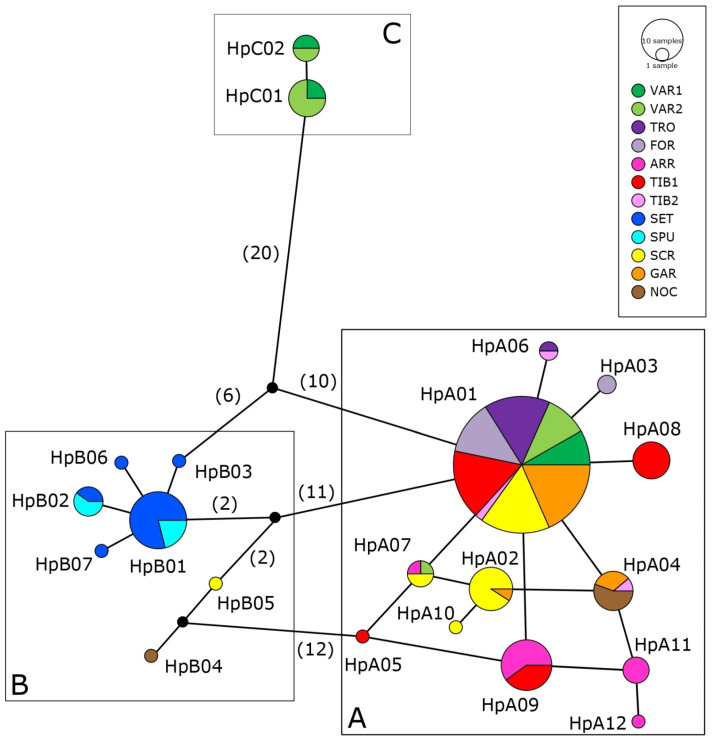
Haplotype network based on CR sequences. Each circle corresponds to one haplotype and its dimension is proportional to the haplotype frequency. The number of nucleotide substitutions between haplotypes is indicated in parenthesis. Boxes highlight the three haplogroups found in this study (A, B and C, see Results). Population abbreviations refer to Table 1.

We identified 21 CR haplotypes from 208 Italian specimens (GenBank accessions: OM966233-53, Figure 3), whose spatial distribution mirrored that observed with COI. Haplogroup A (12 haplotypes) is the most widespread of these and is present in 10 out of 12 Italian sampling sites, from north to south, both in Tyrrhenian and Adriatic slopes; haplogroup B (7 haplotypes) is present in south Tyrrhenian slope, fixed in SET and SPU sites, and rare in SCR and NOC (one specimen each), where it coexists with haplogroup A; haplogroup C (2 haplotypes) is limited to VAR sites, always found together with haplogroup A but less abundant (representing 31% and 40% in VAR1 and VAR2, respectively) (Figure S3). As for COI, a high number of mutational steps was observed between CR sequences belonging to haplogroup C and the most frequent haplotypes of haplogroup A and B: 30 between HpC01 and HpA01, 27 between HpC01 and HpB01; additionally, sequences of haplogroup C were one base shorter (929 bp) than those of the other two haplogroups (930 bp) (Table S3b).

The CR haplotype network (Figure 3) showed a star-like topology for both haplogroup A and B (the latter mainly due to SET specific haplotypes), with few mutations at the intra-haplogroup level (1–4 in A, 1–7 in B, 1 in C). HpA01 is the most common haplotype, being shared by individuals from different basins and slopes; seven haplotypes are shared by individuals from different basins (HpA02, HpA04, HpA06, HpA07, HpA09, HpB01 and HpB02), and others within a single basin (e.g., HpC01 and HpC02 in VAR sites). Haplotype and nucleotide diversity for each site (Table 1) showed the lowest values of Hd in sites from the Adriatic slope (TRO, FOR) and in two of the southernmost ones (GAR, NOC), which also showed the lowest values of π% and haplotype richness, except for NOC, where haplogroup A and B coexist. Higher values of π% were observed in VAR sites, where haplogroup A and C coexist. When calculations were repeated for each haplogroup, similar parameters were obtained for haplogroups A and B, and lower values for haplogroup C, which included the smallest number of samples (Table 2).

Data from the mismatch distribution analysis for all samples did not agree with the expectation of demographic expansion of a homogeneous population; conversely, three different peaks were observed following the presence of the three identified haplogroups (Figure S4). The analysis was then repeated for each haplogroup and a unimodal distribution was obtained for haplogroup A and B. Tajima’s D and Fu’s F consistently has negative values (indicating population size expansion); however, when considering all demographic parameters together, only those referring to haplogroup B agreed with the expansion hypothesis, i.e., it had a significant Tajima’D and R2, θ_1_ equal to 99,999 (Table 2) and the times of demographic growth for B corresponded to 20-9 Kya. No clear pattern was obtained for the small (12 individuals) haplogroup C.

### 3.3. Genetic Structure and Phylogeography

**Figure 4:**
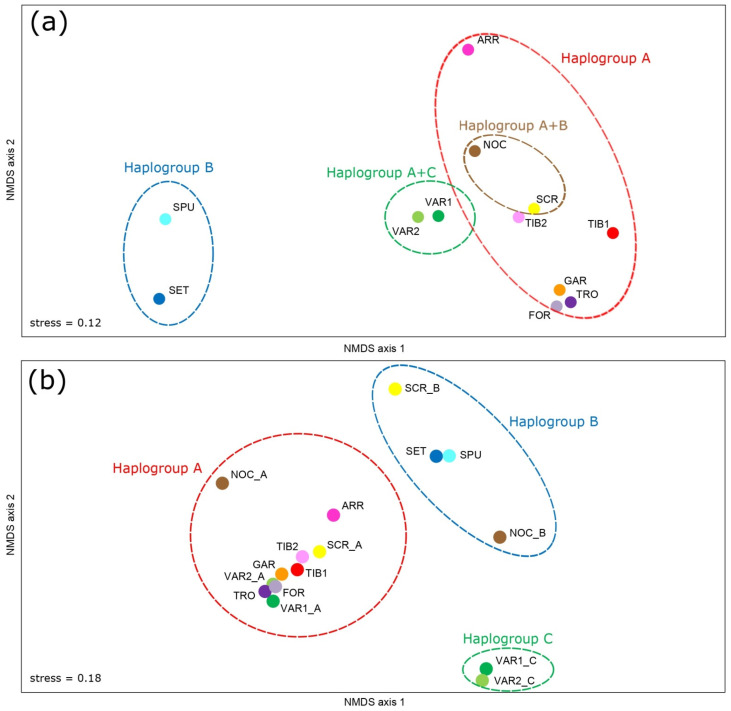
Non-metric multidimensional scaling based on CR matrix of ΦST on (**a**) sampling sites, (**b**) splitting individuals by haplogroup when two haplogroups occur in the same site (in VAR, SCR, NOC). Populations colors and abbreviations as in Figure 1.

Global ΦST (0.577, *p* < 0.001) indicated high genetic differentiation among sites. Calculation of pairwise ΦST values (Table S6a) showed that the highest significant values were observed in comparisons involving sites with a homogenous composition, i.e., between B-only and A-only sites (0.95 < ΦST < 0.98); however, the results were partly biased at sites where individuals belonging to different haplogroups were present (e.g., VAR). It is noteworthy that significant values were also obtained among sites within haplogroup A (e.g., see ARR, TIB1). To deepen the analysis on genetic differentiation between the main haplogroups and sites, pairwise ΦST calculation was repeated by separating the different haplogroup components, when they occur in the same site simultaneously (in VAR, SCR, NOC) (Table S6b). Values obtained between different haplogroups were high (0.93 < ΦST < 1.00) but not always significant, likely due to a very small sample size. NMDS plots were obtained by considering all the sites (Figure 4a), and the main haplogroups within the sites (Figure 4b) allowed us to identify the main haplogroups pattern and further subdivisions within the most represented ones (haplogroups A and B). Indeed, within haplogroup A, all populations were placed together but NOC_A and ARR appeared to be separated from the others, similarly to haplogroup B, where SCR_B and NOC_B were separated from SET/SPU but in different directions so that three B subgroups were graphically identified.

Among the different hypotheses tested using AMOVA, statistical support was obtained when considering subdivision with three haplogroups, with 95.06% of the variation explained by among-groups differences (Table 3). When a further subdivision within haplogroups, suggested by NMDS, was tested, a similar among-groups percentage was observed (95.18%) but also a smaller value for within-groups variation. On the contrary, a subdivision of samples purely based on ichthyogeographic district origin did not provide statistically supported results.

**Figure 5:**
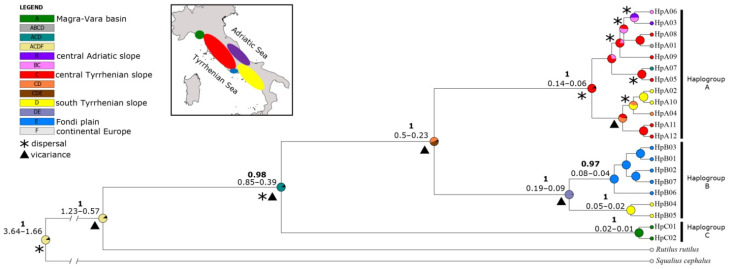
Bayesian CR tree with time estimates at supported nodes and ancestral area reconstruction of Sarmarutilus haplogroups and haplotypes. Posterior probability higher than 0.90 in bold; time estimates are in Mya; * denotes dispersal events, triangle vicariance events. Area reconstructions lower than 15% are indicated in black as null.

Genetic distances between haplogroups were higher when comparing C to A and B (0.033 and 0.029, respectively, Table S5), than between A and B (0.015). A dating analysis and ancestral areal reconstruction (S-DIVA) based on the CR BI tree revealed a complex phylogeography for *Sarmarutilus* in Italy (Figure 5). Vicariance events were estimated at the basal split of haplogroups; the divergence time of lineage C corresponded to 850–390 thousand years ago (Kya) while between A and B it corresponded to 500–230 Kya. Within haplogroup A, the S-DIVA analysis identified many dispersal events, despite nodes not being supported, from the central to south Tyrrhenian slope and Adriatic slope. Within haplogroup B, S-DIVA estimated a vicariance event between the Fondi plain and the south Tyrrhenian slope.

## 4. Discussion

In this paper, the analysis of mitochondrial DNA in Italian and Albanian roaches, complemented with sequences from representatives of all the currently recognized *Rutilus* and *Leucos* species, confirmed a loose relationship between the two investigated taxa, despite the very similar morphology. Our phylogenetic reconstruction also highlighted the need for an in-depth taxonomical revision of Balkan roach species. Finally, our intraspecific analysis revealed a deep and complex phylogeographic structure for Italian *S. rubilio*, masked by secondary contacts between lineages.

### 4.1. Molecular Identification and Phylogeny

The COI molecular identification and phylogenetic analysis provide new insights into the relationships among roach species. Our analysis was overall congruent with the COI tree obtained in previous studies [72]; however, due to the integration of a higher number of sequences from different basins and the molecular identification of new lineages, more details emerged. Despite this, some main points remain unresolved.

The Albanian specimens examined in this study (from Skadar and Ohrid lakes, morphologically similar to Italian *S. rubilio*) were clustered separately from Italian samples in the phylogenetic tree. Specifically, specimens from each lake showed one to three site-specific mutations, and haplotypes from the Skadar lake clustered with *L. albus* (typical from this lake) while those from the Ohrid lake grouped with *L. basak* sequences from this site (recorded in GenBank as *R. ohridanus*); furthermore, the Ohrid lake cluster also included *L. aula* and *L. basak* from lake Prespa in Albania (recorded in GenBank as *R.prespensis*). The status of the *Rutilus*/*Leucos* species of the Balkan area is debated and there is no agreement on the validity and distribution of the previously mentioned species. Indeed, based on their morphological and genetic traits, the following three species were identified [37]: *Rutilus albus* in Lake Skadar, *R. ohridanus* endemic in the Ohrid lake, and *R. prespensis* present both in Lake Prespa and Lake Skadar (in this site in sympatry with *R. albus*). Subsequently, the taxonomy of the genus *Rutilus* was revisited [30], wherein the genus *Leucos* was resurrected, and in the Adriatic Balkan area, *R. albus* was renamed *Leucos albus.* Moreover, *R. basak* from the Neretva basin (between Croatia and Bosnia-Herzegovina) was named *Leucos basak*, and the Albanian taxa distributed in the Ohrid lake (previous *R. ohridanus*), Prespa lake (previous *R. prespensis*) and Skadar lake (*R. prespensis* in sympatry with *L. albus*) were considered synonyms of *L. basak* [73]. Our data disagree with this attribution, as the non-monophyletic status of current *L. basak*. specimens from Neretva are highly divergent from those from the Albanian lakes included in this taxon. Indeed, the Neretva and Albanian basins belong to different ichthyogeographic districts [74]. Conversely, specimens from the Ohrid and Prespa lakes are grouped with *L. albus* in the same monophyletic clade according to previous reports [25]. These data allow us to draw some conclusions on the taxonomy of these species. First, Albanian taxa cannot be considered synonyms of *Leucos basak* from the Neretva basin. Second, the choice to synonymize *Rutilus prespensis*, *R. ohridanus* and *R. albus*, considering their close genetic relationship [20], is supported by our data. This seems a wise parsimony choice that fits the need to avoid the taxonomy inflation that characterizes other fish taxa [75], and also considers the low number of mutations between the haplotypes collected in Ohrid or Skadar lakes. Meanwhile, diagnostic mutations are present between fishes of these lakes, and this differentiation should not be disregarded, as they can potentially be considered as geographic populations. Moreover, morphological differentiation is reported between *L. albus* and the sympatric taxa in the Skadar lake and further integrated analyses identifying morphological characteristics, multiple genetic markers and environmental parameters are needed to clarify the basis of such diversity.

Another problematic issue involves *Rutilus virgo*. Our COI phylogenetic analysis clustered this taxon into the *Leucos* genus with strong support, according to previous research [25,76]. However, breeding males of this species show horizontal rows of large tubercles on their scales and head [39] that are typically absent in the *Leucos* species [30]. In addition, some zoogeographic features differentiate *R. virgo* from the *Leucos* species, including the following: the former inhabits rivers flowing into the Danube River–in Slovakia, and Slovenia, rarely in the Czech Republic [77] while *Leucos* species are typically present in rivers of the Balkans and Italy flowing in the Adriatic Sea. Thus, the relationship between *R. virgo* and the other roach species remains ambiguous. Despite *Leucos* being a well-supported monophyletic clade, its relationship with other *Rutilus* species and *Sarmaratuilus* were not resolved in our analysis; as in previous studies [25,72], we were unable to define whether *Leucos* or *Sarmarutilus* are sister groups of *Rutilus* or, conversely, if they are included in *Rutilus*, so that the latter is not monophyletic. This issue will need to be addressed in the future through analyses based on a combined dataset for all roach species. Finally, our tree topology lends support to the differentiation of the endemic roach from the Volvi lake and Struma river in Greece, as the COI sequences from these sites retrieved from GenBank (there labelled as *R. heckelii* and *R. lacustris*, respectively) are grouped in a well-supported cluster separated from that including *R. lacustris* from Black and Caspian Sea basins (one *R. rutilus* sequences clustering into *R. lacustris* may be due to misidentification as the two species are sympatric in Eastern Europe). Roaches from Volvi lake were recognized as a distinct species, *Rutilus stoumboudae*, based on both their morphological and molecular characteristics [30,78]. However, this putative species is currently considered a synonym of *R. lacustris* [73,79], due to the low genetic divergence from this species. Our data confirm this taxonomic attribution, and as for the case of *R. orhidanus*/*prespensis*, we suggest considering Volvi roach specimens as a geographic population that has peculiar genetic traits allowing its distinction from *R. lacustris* from the Ponto Caspian area. New studies covering other Greek localities will clarify its distribution in Greece and its conservation status, as the population is probably extinct in the Volvi lake [30].

### 4.2. Divergence between Sarmarutilus Lineages: Cryptic Species?

The COI phylogenetic tree and both COI and CR haplotype networks revealed three different haplogroups among Italian specimens, all of which were morphologically identified as *Sarmarutilus rubilio*. Two haplogroups (A and B) matched with sequences previously identified by other authors [20], while the third one (C), exclusive to the Magra-Vara basin (VAR sites), was never recorded before and did not show adequate similarity with any sequence stored in international databases (GenBank, BOLD). COI divergence obtained for the lineage C ranges from 3.3 to 2.9% (A–C and B,C haplogroups pairwise comparison, respectively). According to what was observed in 1088 fish species [80], sequences with a divergence of greater than 2% or 3% likely belong to different species (with a probability greater than 95%). This COI divergence threshold was generally confirmed with an analysis implementing several methods for species delimitations in fishes and allowed for the identification of cryptic species [81,82,83] even in Leuciscidae [84]; furthermore, 2–3% divergence was observed between many *Leucos* and *Rutilus* taxa whose status as good species is not debated. Although our nuclear dataset is limited, a substantial divergence (almost 4%) and a slight difference in length were observed between Cyfun P sequences exclusive to VAR sites and mostly present in specimens belonging to mitochondrial lineage C, and those widespread across *S. rubilio* populations. In other Leuciscidae species, no intraspecific variability in Cyfun P sequence length was observed [42], and a very small divergence (0.3–1.5%) or none at all (if recently split) was observed between species [4,85]. These data are highly indicative of typical Magra-Vara lineage C being a putative new cryptic *Sarmarutilus* species. More in-depth investigations, including a wider nuclear dataset, are necessary to strengthen confidence in our hypothesis. These roaches coexist with lineage A and, as specimens with various combinations of nuclear and mtDNA lineage were found, they can hybridize. This commonly happens in Leuciscidae, between different species and even genera [86,87,88], and genetic introgression was also reported, not always associated with intermediate morphological characters [4,85,89]. In the absence of morphological diagnostic traits (see Results), a formal description of this putative new species is currently not possible. Moreover, we are carrying out a geometric morphometrics analysis for all Italian specimens to investigate the morphological differences at a finer scale, and the results will be displayed and discussed in a new paper. Further sampling is also needed to map the distribution of C lineage in the Magra-Vara basin and in nearby small catchments that could also host yet undetected *Sarmarutilus* populations.

### 4.3. Phylogeography of Italian Sarmarutilus

The presence of multiple haplogroups within *Sarmarutilus* and their coexistence in some sampling sites are congruent with the hypothesis of multiple refugia in Italy during Pleistocene glacial cycles and subsequent secondary contact, as observed in other freshwater taxa [90,91,92,93,94]. Estimated time splits between *Sarmarutilus* haplogroups (850 to 230 Kya) indeed correlate with Pleistocene events. In addition to past climatic fluctuations, freshwater fishes’ phylogeographic patterns and distribution in Italy were also driven by mountain ridges and the presence/lack of connections among drainages [14,27,95]; this is especially true for those taxa, such as Leuciscidae, that cannot disperse through the sea due to their little or no tolerance to brackish water [26] and references therein. Indeed, the Apennine chain, dividing peninsular Italy longitudinally from north to south, was found to influence the biogeographic structure even of semiaquatic vertebrate species but not that of the peninsular terrestrial ones (if not marginally) [96].

Despite this, the current distribution of *Sarmarutilus* haplogroups only partially matches the currently recognized ichthyogeographic Italian districts, which are characterized by different hydrogeological histories and evolution and are inhabited by different species [21,97]. See details on each haplogroup below.

#### 4.3.1. Haplogroup C

The presence of the most ancient lineage (haplogroup C) only at the northern border of *Sarmarutilus* range (Magra-Vara basin), permits some inferences on the origin of this genus.

There is no record of roaches westward to this basin, while northward, typical PV district roach species are present, i.e., *Leucos aula* and *Rutilus pigus*, are not strictly related to *Sarmarutilus* (see Figure 2). The Magra-Vara basin has been geographically isolated since the late-Pliocene and early Pleistocene tectonic uplift of the surrounding mountains as follows [98]: (a) from the PV district by the northern Apennines, thus representing the northern border of most of the primary freshwater fish species native to Central Italy [99]; (b) from southern catchments by the Apuan Alps, a geographic barrier extending from the main Apennine to the sea which greatly effects the genetic structure of freshwater species [26,28,100]. In the early Pleistocene, when most of the current major southern basins such as Arno and Tiber were limited in extension due to marine introgression, Magra-Vara still had connections to both basins flowing in the Tyrrhenian Sea and the Adriatic Sea [98]. Thus, it is likely that *Sarmarutilus* moved initially from the Balkans (where it originated, see Introduction) to Magra-Vara through brackish waters [96] and from here it spread to southern catchments. This colonization path is in contrast to the immigration route observed in other Italian vertebrates [96] including other leuciscidae [15,97], i.e., from the Balkans to south Italy and a subsequent northward expansion. The split between *Sarmarutilus* lineage C and *S. rubilio sensu stricto* can be dated back to 850–390 Kya, supporting the hypothesis that the uprising of the Apuan Alps in the early Pleistocene promoted the isolation of lineage C in this basin from *S. rubilio* present in southern catchments.

The presence of the widespread *S. rubilio* lineage A in Magra-Vara today will be discussed later.

A demographic analysis of haplogroup C indicated no deviance from a neutral/stable population and thus the absence of post-glacial population expansion, as conversely observed in this site for *Squalius lucumonis* [28]. Currently, we are not able to decipher whether lineage C demography was unaffected by the alternation of glacial and interglacial periods or if the secondary contact (and partial hybridization) with *S. rubilio* haplogroup A caused the depletion of lineage C haplotypes. Haplogroup A is the most frequent in both Magra-Vara sites and some vantage selection may exist (see Haplogroup A discussion, here below).

#### 4.3.2. Haplogroup A

This haplogroup is the most widespread, present from the northern to southern distribution limits and in most of the sites and is typical in the TL district, but also present in the AC and the Adriatic slope of Central Italy.

Haplogroup A originated in the TL district after the first split from lineage C and a second one from B, during the Pleistocene, i.e., when it likely remained isolated during the glacial phase somewhere in a refugium and underwent allopatric divergence; after that, dispersal events promoted its geographic spread through the Apennine chain, as suggested by S-DIVA (Figure 5). Indeed, these mountains, despite isolating the Tyrrhenian and Adriatic slopes of Central Italy and being considered the natural border between TL and PV districts and between TL and the northern Adriatic section of AC, could have acted as a semi-permeable barrier. This seems plausible especially for rheophilic species [101], through river connections acting in the upper section of basins [102], and as already reported in Mediterranean trout [103,104]. *Sarmarutilus rubilio* is not a strict rheophilic species; however, it is found in Apennine rivers and could have spread through the hydrographic network, which could explain the presence of HpA01 in the Adriatic slope (TRO and FOR) and the high differentiation observed in those sites that are not linked to the Apennines, i.e., ARR drainage that is fed by the Bracciano volcanic lake [105]. Their existence along the entire Apennine chain of the intermontane lakes up to the Late-Pleistocene [106,107] could also have had an important role in haplogroup A dispersion [108].

The presence of the most common haplotype HpA01 in Magra-Vara, co-existing with lineage C, may be the result of secondary contact. Since *S. rubilio* can adapt to nearly all water habitats, from lentic to lotic, from nearby sea to 1250 m a.s.l. [34], past sea-level changes could have allowed the re-colonization of Magra-Vara from southern catchments through mouth river confluences, a dispersion route observed in freshwater fishes that inhabit coastal streams [109]. Such migration should have occurred quite recently, during the last glacial age (about 22 Kya), when the sea level was lower than today [110,111]. Alternatively and in addition, a human-driven translocation of lineage A in the Magra-Vara basin could not be excluded, but it is unlikely as all allochthonous species present in this basin are those typical of the northern PV district [99], suggesting the translocation of fishes only from geographic areas where *S. rubilio* is absent. Mitochondrial lineage A, besides being the most widespread, is also the most frequent when coexisting with the others (Figure S3); therefore, we cannot exclude a higher fitness associated with lineage A and/or some kind of competition when secondary contact occurs, as suggested in other freshwater fishes when different lineages come in contact [104].

Neutrality tests and mismatch analysis values suggested demographic expansion when considering all individuals belonging to the widespread haplogroup A, although these results were not fully statistically supported. The combination of Hd and π% values for the main TL district sites (ARR and TIB1-2, Hd > 0.5 and π < 0.5%), and those outside TL where only A was found (TRO, FOR and GAR, Hd < 0.5 and π% < 0.05) provided different estimates, suggesting a population bottleneck followed by rapid population growth for the former and a recent population bottleneck or founder event for the latter [112].

Finally, considering both the hydrogeological history of Italy and genetic data of *S. rubilio*, it is likely that haplogroup A experienced demographic growth after the glacial period within the TL district and a recent natural range expansion through the Central and Southern Apennines; conversely, translocation events occurred southward to the species’ native range [113,114], and likely involved individuals from the TL district, thus explaining the presence of only haplogroup A in the southernmost CRA site (Figure 1 and Figure 2).

#### 4.3.3. Haplogroup B

Haplogroup B is present in the area including the Fondi plain (SET, SPU) where the lineage is fixed; in Liri-Garigliano surroundings (SCR) not attributed to either TL or AC districts [21]; and also over the southern border of AC (NOC). In the latter sites, lineage A is also present. The split between haplogroup A and B was estimated at 500–230 Kya, which is congruent with the emergence of the Colli Albani Volcano (around 450–400 Kya) on the Tyrrhenian southern border of the TL district. The volcano reversed the flow of rivers currently belonging to the Liri-Garigliano drainage from the north (toward the Tiber basin, TL district) to the south [115,116], thus likely breaking the connection between Central and Southern Italy and initiating allopatric genetic differentiation. After that, haplogroup B differentiated into subclusters. The Fondi plain was never reached by haplogroup A, likely due to the isolation of local basins from north to east by the Ausoni-Aurunci mountains, whose uplift started in the Pliocene [117], and from west to south by the sea. Conversely, in the nearby Liri-Garigliano area (about only 30 km far from Fondi plain) and southward, the main rivers originated in the Apennines and secondary contact occurred.

Neutrality tests and demographic parameters supported the demographic expansion hypothesis for haplogroup B, with the main contribution provided by Fondi plain populations (SET, SPU). In these sites, several B haplotypes were connected by the haplotype network in a typical star-like shape, due to the few mutations between each other. Demographic expansion can be explained by considering recovery after the glacial period, and thus accounting for individuals spreading from glacial refugia after harsh climate conditions, as already observed in other freshwater fishes [18,28]. Time since expansion for haplogroup B was estimated at 20 and 9 Kya, while when recalculating for SET and SPU it was only was 19 and 8 Kya, which is subsequential to the Last Glacial Maximum (22 Kya). Thus, the Fondi plain could likely represented not only an area of isolation from widespread haplogroup A but also a glacial refugium.

The distribution of lineage B also in the area between the Tyrrhenian northern border of AC and the southern one of TL, i.e., Liri-Garigliano and the Fondi plain (and northward to SIS site, see Figure 1), agrees with those of other freshwater fishes typical of the AC district, such as the barbel *Barbus fucini* [97], and the loach *Cobitis zanandreai* [118]. We suggest that the Tyrrhenian upper border of the AC district should be moved northward, close to the Tiber river mouth, thus including the aforementioned area. On the Adriatic slope, results from the two sites investigated did not allow us to identify the border between the AC and the other districts; moreover, the distribution of other south Italy endemic species in this area, such as *Barbus samniticus* [97], suggests movement of the border on this side further north to the Vomano basin, thus including our FOR site.

### 4.4. Implication for Conservation and Management

From a management perspective, to prevent the local extinction of *Sarmarutilus* populations and preserve their genetic uniqueness, the following three main points emerged from our analysis:

(a). New research is necessary to precisely map the distribution of lineage C. This new putative cryptic species is endemic to the Magra-Vara basin and is found in tributaries not included in protected areas; therefore, it could be particularly exposed to threats. This underlines the importance of also protecting small river course habitats, as they might represent refugia of relict native fish populations [119].
(b). Conservation actions required by the Habitats Directive for the South European roach [35] should consider that haplogroups A and B represent two conservation units, namely Evolutionary Significant Units (ESUs, for a synthesis of this definition, see [120]) and as such should be managed.
(c). The main conservation efforts should be focused on avoiding the introduction of allochthonous species, especially those that can compete or hybridize with *Sarmarutilus*. In addition, the translocation of *Sarmarutilus* populations from different districts and within the AC district (where subclusters are present) should be avoided, as these roaches represent different genetic entities.

## 5. Conclusions

Our phylogenetic analysis of roach species assigned all specimens from peninsular Italy to the genus *Sarmarutilus*, while Albanian ones were assigned to the *Leucos genus*. We highlighted the need for further in-depth analyses of roaches from Albanian lakes, as their current identification as different species (*L. allbus* and *L. basak*) was not well supported, and *L. basak* was revealed to be a paraphyletic taxon. We also suggest focusing on other Balkanian taxa in future as relationships between species and the genetic distinctiveness of some populations have yet to be fully understood.

A significative intraspecific difference within South European roach was already noticed [20] (Appendix S1), and the need for further analyses and a taxonomical update was suggested. Our phylogeographic analysis confirmed this idea by revealing a strong genetic structure with a strict geographic basis across peninsular Italy, suggesting a long history of isolation. Pleistocene vicariance events, due to the emergence of geographic barriers such as mountainous ridges and volcanoes, promoted allopatric genetic divergence among the three identified haplogroups, of which C could probably be considered as a new putative cryptic species. This latter species, despite the high genetic divergence, lacks morphological/meristic distinctive features; therefore, we are currently working on geometric morphometrics to detect differences in shape which could differentiate the two species. Haplogroups A and B underwent different phylogeographic histories, whereby A originated in Central Italy, subsequently spread across the whole areal and came into secondary contact with the other haplogroups, while B originated in the Tyrrhenian slope of Central-Southern Italy, where it is currently restricted, and likely experienced further allopatric diversification.

New data are needed to thoroughly assess the extent of *S. rubilio* genetic diversity, from uninvestigated areas of native ranges and other nuclear molecular markers.

## Figures and Tables

**Table 1:** Sampling details for twelve populations of South European roach from Italy and two *Rutilus* sp. from Albania and summary of genetic variation for the mitochondrial control region (CR) and cytochrome oxidase I (COI) subset.

							CR				COI	
Drainage Basin	River/Lake	Pop ID	Lat (°N)	Lon (°E)	Protected Area	N	Hp (% Private)	Hp Rich	Hd (±s.e.)	π % (±s.e.)	N	Hp
Italy												
Magra-Vara	Riccò	VAR1	44.161	9.758		13	3 (0)	3.00	0.51 (±0.14)	1.51 (±0.81)	11	4
	Graveglia	VAR2	44.191	9.790		20	4 (0)	3.54	0.63 (±0.08)	1.65 (±0.86)	8	4
Tronto	Tronto	TRO	42.802	13.465	SAC	18	2 (0)	1.72	0.11 (±0.10)	0.01 (±0.02)	3	2
Foro	Foro	FOR	42.246	14.186		16	2 (50)	1.98	0.23 (±0.13)	0.03 (±0.03)	3	2
Arrone	Arrone	ARR	41.914	12.265		15	4 (50)	3.73	0.60 (±0.11)	0.09 (±0.08)	3	2
Tiber	Rio Martino	TIB1	42.173	12.545		33	4 (50)	3.35	0.63 (±0.06)	0.08 (±0.07)	2	2
	Fosso Passerano	TIB2	41.932	12.732		4	3 (0)	NA	0.83 (±0.22)	0.11 (±0.11)	2	2
Fondi	Settecannelle	SET	41.368	13.421	Regional Park	20	5 (60)	3.84	0.44 (±0.13)	0.05 (±0.05)	3	2
San Puoto	San Puoto	SPU	41.285	13.408	SPA	7	2 (0)	NA	0.57 (±0.12)	0.06 (±0.06)	7	1
Santa Croce	Santa Croce	SCR	41.287	13.716	SAC	32	5 (40)	3.47	0.60 (±0.07)	0.21 (±0.13)	3	2
Liri-Garigliano	Ausentello	GAR	41.303	13.743		24	3 (0)	2.46	0.30 (±0.11)	0.04 (±0.04)	3	1
Noce	Pamafi	NOC	39.934	15.752		6	2 (50)	NA	0.33 (±0.22)	0.58 (±0.38)	6	2
Total						208	21				54	14
Albania												
Skadar	Skadar	SKA	42.059	19.455		8	8 (100)	NA	1.00 (±0.06)	0.39 (±0.25)	8	4
Ohrid	Ohrid	OHR	40.963	20.640		7	4 (100)	NA	0.71 (±0.18)	0.12 (±0.1)	7	3
Total						15	12				15	7

Abbreviations: sample size (N), number of haplotypes and percentage of private haplotypes (Hp), haplotype richness (Hp rich), haplotype diversity (Hd), nucleotide diversity (π). Standard errors (s.e.) are given in parentheses for Hd and π. SAC = Special Area of Conservation, according to EC Habitat Directive; SPA = Special Protection Area, according to EC Birds Directive. Datum = WGS84 for geographic coordinates.

**Table 2:** Summary of genetic variation, neutrality test and mismatch distribution parameters for the mitochondrial control region (CR) for each haplogroup and all samples.

Haplogroup	N	Hp	Hd (±s.e.)	π % (±s.e.)	Tajima’s D	Fu’s F	R2	SSD	Hri	τ	θ_0_	θ_1_
A	167	12	0.56 (±0.04)	0.09 (±0.07)	−0.919	−6.561 *	0.052	0.001	0.056	0.926	0.100	3.196
B	29	7	0.55 (±0.10)	0.13 (±0.09)	−1.722 *	−2.237	0.062 **	0.014	0.119	0.725	0.000	99,999.000
C	12	2	0.48 (±0.11)	0.05 (±0.05)	1.066	1.003	0.242	0.019	0.236	0.734	0.000	99,999.000
Tot.	208	21	0.71 (±0.03)	0.75 (±0.39)	−0.107	1.541	0.081	0.035	0.040	0.072	1.816	99,999.000

Abbreviations: sample size (N), number of haplotypes (Hp), haplotype diversity (Hd), nucleotide diversity (π), standard errors (s.e.), sum of squared deviations (SSD), Harpending’s raggedness index(Hri), time since expansion in mutation units (τ), population size estimators before and after the expansion (θ_0_ and θ_1_). Significance thresholds: * *p* < 0.05; ** *p* < 0.01.

**Table 3:** AMOVA hierarchical analysis for Italian specimens, examining the partitioning of genetic variance of the mitochondrial control region (CR).

	Among Groups		Among Populations within Groups		Within Populations	
N. of Groups and Group Composition	%var	ΦCT	%var	ΦSC	%var	ΦST
(1) no structure: 1 group	--	--	57.68	--	42.32	0.57682 ***
(2) ichthyogeographic districts: 3 groups (PV = TRO; TL = VAR1, VAR2, ARR, TIB1, TIB2; AC = FOR, SET, SPU, SCR, GAR, NOC)	−9.87	−0.09873	66.04	0.60108 ***	43.83	0.56169 ***
(3) Haplogroups: 3 groups (HpC = VAR1_C, VAR2_C; HpA = VAR1_A, VAR2_A, TRO, FOR, ARR, TIB1, TIB2, SCR_A, GAR, NOC_A; HpB = SET, SPU, SCR_B, NOC_B)	95.06	0.951 ***	2.07	0.418 ***	2.88	0.971 ***
(4) NMDS, subgroups within haplogroups: 7 groups (VAR1_C, VAR2_C; VAR1_A, VAR2_A, TRO, FOR, TIB1, TIB2, SCR_A, GAR; ARR; NOC_A; SET, SPU; SCR_B; NOC_B)	95.18	0.952 ***	0.87	0.181 ***	3.95	0.961 ***

Hypothesized structures: (1) no structure (one group), (2) subdivision by ichthyogeographic district (three groups), (3) subdivision by haplogroup (three groups), (4) clusters identified by NMDS analyses (seven groups). Significance thresholds: *** = *p* < 0.001.

## Data Availability

Sequences were deposited in the GenBank database (NCBI) with the accession numbers: OM974277- OM974297 (COI), OM966233-OM966253 (CR), OM966266-OM966281 (Cyfun P), OM966282- OM966297 (S7). All vouchers (AC1687-AC1698) are deposited in the Museum of Comparative Anatomy “Battista Grassi”, Department of Biology and Biotechnology “Charles Darwin”, Sapienza University of Rome.

## Supplementary Materials

The following supporting information can be downloaded at: https://www.mdpi.com/article/10.3390/genes13061071/s1, Table S1: PCR conditions and primers for each amplified marker [121,122,123], Table S2: COI Sequences retrieved from GenBank and BOLD, Table S3: COI (a) and CR (b) diagnostic sites identified among the most frequent sequences of the three haplogroups, Table S4: Diagnostic fragment of nuclear marker Cyfun P, Table S5: Inter- and intra-haplogroup genetic mean distances; pairwise ΦST between haplogroups, Table S6: Population ΦST (CR) considering all the populations as they are (a) and splitting individuals by haplogroup for each one (b), Figure S1: COI haplotype network, Figure S2: Specimens with mtDNA and Cyfun P belonging to lineage C (a) and lineage A (b), Figure S3: Spatial haplogroups distribution in *S. rubilio*, Figure S4: mismatch distributions of CR sequences.
