# Supplementary material for: Phylogeography of Sarmarutilus rubilio (Cypriniformes: Leuciscidae): Complex Genetic Structure, Clues to a New Cryptic Species and Further Insights into Roaches Phylogeny
Source: Genes (Basel). 2022 Jun 15;13(6):1071. doi: 10.3390/genes13061071 (PMC9222716; doi:10.3390/genes13061071)
Supplement: Supplementary file 1 [file genes-13-01071-s001.zip › genes-1734495-supplementary.pdf]

Supplementary Material for

Phylogeography of Sarmarutilus rubilio (Cypriniformes: Leuciscidae): complex genetic structure, clues to a new cryptic species and further insights into roaches phylogeny

Gerardo Petrosino [1,*], Lorenzo Tancioni [2], Martina Turani [1], Arnold Rakaj [2], Luca Ciuffardi [3], and Anna Rita Rossi [1]

1 Department of Biology and Biotechnology "C. Darwin", Sapienza University of Rome, 00185 Rome, Italy; gerardo.petrosino@uniroma1.it (G.P.); annarita.rossi@uniroma1.it (A.R.R.)
2 Department of Biology, University of Rome "Tor Vergata", 00133 Rome, Italy; tancioni@uniroma2.it (L.T.); arnoldrakaj@gmail.com (A.R.)
3 Center for BioNatural Studies srl, 16132 Genova, Italy; info@lucaciuffardi.it
*** Correspondence: gerardo.petrosino@uniroma1.it**

Table S1. PCR conditions and primers for each amplified marker and PCR product length. Total volume reaction = 20 µl: 2 µl of 10x NH_4 Buffer, 0.6 µl of 50 mM $MgCl_2$, 0.4 µl of dNTP mix (2.5 µM each dNTP), 0.2 µl of 100 µM of each primer, 0.14 µl of 5 $U\mu l^{-1}$ of BIOTAQ™ DNA Polymerase (Bioline) and 10–100 ng of template DNA.

Marker	Primer sequence	Reference	PCR conditions	Gene length (bp)
COI	FISHF1: 5'TCAACCAACCACAAAGACATTGGCAC3'<br>FishR2 5'-ACTTCAGGGTGACCGAAGAATCAGAA-3'	[121]	95ºC, 2 min - 30 cycles: 94ºC, 30 s; 54ºC, 30 s; 72º, 1 min - 72ºC, 10 min.	624
CR	ESTFOR: 5'CATCGGTCTTGTAATCCGAAGAT3'<br>PHE1R: 5'ACATCTTCAGTGTTACGCTT3'	[122]	95ºC, 2 min - 30 cycles: 94ºC, 30 s; 54ºC, 30 s; 72º, 1 min - 72ºC, 10 min.	929–930
Cyfun P	Cyp_un FLP1: 5'AAGTGGTGCATCGTGTTGTG3'<br>Cyp_unFL1R: 5'CAGCCTGAACAATCAAAACAG3'	[42]	94ºC, 3 min - 35 cycles: 94ºC, 30 s; 55ºC, 30 s; 72º, 1 min - 72ºC, 10 min.	156–218*
S7	S7RPEX1F: 5'TGGCCTCTTCCTTGGCCGTC3'<br>S7RPEX2R: 5'AACTCGTCTGGCTTTTCGCC3'	[123]	95ºC, 3 min - 30 cycles: 95ºC, 30 s; 54ºC, 1 min; 72º, 2 min - 72ºC, 10 min.	319–861*

*total alignment including gaps

References

121. Lieckfeldt, D.; Hett, A.K.; Ludwig, A.; Freyhof, J. Detection, Characterization and Utility of a New Highly Variable Nuclear Marker Region in Several Species of Cyprinid Fishes (Cyprinidae). *Eur. J. Wildl. Res.* **2006**, *52*, 63–65. https://doi.org/10.1007/s10344-005-0010-6.
122. Ward, D.R.; Zemlak T.S.; Innes B.H.; Last P.R.; Hebert, P.D.N. DNA barcoding Australia's fish species. *Philos. Trans. R. Soc. B*, **2005**, *360*, 1847–1857. https://doi.org/10.1098/rstb.2005.1716.

123. Gilles, A.; Lecointre, G.; Miquelis, A.; Loerstcher, M.; Chappaz, R.; Brun, G. Partial Combination Applied to Phylogeny of European Cyprinids Using the Mitochondrial Control Region. *Mol. Phylogenetics Evol.*, **2001**, *19*, 22–33. https://doi.org/10.1006/mpev.2000.0916.
124. Chow, S.; Hazama, K. Universal primer for S7 ribosomal protein gene intron in fish. *Mol. Ecol.*, **1998**, *7*, 1255–1256.

Table S2. COI Sequences retrieved from GenBank (https://www.ncbi.nlm.nih.gov/genbank/) and Barcode Of Life Data SYSTEM (www.boldsystems.org, in yellow) included in the phylogenetic reconstruction.

Species label from databases (Genus *Rutilus*)	Accession Number	Current valid species name (Eschmeyer Catalogue of Fishes, https://www.calacademy.org/scientists/projects/eschmeyers-catalog-of-fishes)	Country (basin)	ID site (only *S. rubilio*, see Figure 1)
R. albus	MG806869	*Leucos albus*	Albania (Drin)	
R. albus	KJ554332	*Leucos albus*	Albania (Skadar lake)	
R. albus	KJ554244	*Leucos albus*	Albania (Skadar lake)	
R. albus	KJ554242	*Leucos albus*	Albania (Skadar lake)	
R. aula	KJ554532	*Leucos aula*	Italy (Po)	
R. aula	KJ554171	*Leucos aula*	Croatia (Zadar)	
R. aula	HM560323	*Leucos aula*	Croatia (Bacisca lake)	
R. aula	MG806870	*Leucos aula*	Croatia (Zrmanja)	
R. basak	MG806871	*Leucos basak*	Bosnia and Herzegovina (Neretva)	
R. basak	HM560325	*Leucos basak*	Bosnia and Herzegovina (Neretva)	
R. basak	KJ554498	*Leucos basak*	Bosnia and Herzegovina (Neretva)	
R. basak	KJ554191	*Leucos basak*	Bosnia and Herzegovina (Mandek)	
R. frisii	MG806872	*Rutilus frisii*	Bulgaria (Rezowska)	
R. frisii	KJ554245	*Rutilus frisii*	Bulgaria (Rezowska)	
R. frisii	MT756353	*Rutilus frisii*	Iran (Gorgan Gulf Caspian Sea)	
R. frisii	MT756352	*Rutilus frisii*	Iran (Gorgan Gulf Caspian Sea)	
R. heckelii	KJ554478	*Rutilus lacustris*	Greece (Volvi lake)	
R. heckelii	KJ554441	*Rutilus lacustris*	Greece (Volvi lake)	
R. heckelii	KJ554358	*Rutilus lacustris*	Greece (Volvi lake)	
R. heckelii	KJ554261	*Rutilus lacustris*	Greece (Volvi lake)	
R. kutum	AQM026-16	*Rutilus kutum*	Iran (Mazandaran Province)	
R. kutum	AQM027-16	*Rutilus kutum*	Iran (Mazandaran Province)	
R. kutum	AQM028-16	*Rutilus kutum*	Iran (Mazandaran Province)	

R. kutum	AQM029-16	*Rutilus kutum*	Iran (Mazandaran Province)	
R. lacustris	MT756380	*Rutilus lacustris*	Iran (Gorgan Gulf Caspian Sea)	
R. lacustris	HQ561910	*Rutilus lacustris*	Georgia (Rioni)	
R. lacustris	HQ561909	*Rutilus lacustris*	Georgia (Rioni)	
R. lacustris	MG806873	*Rutilus lacustris*	Greece (Struma)	
R. meidingeri	KR477255	*Rutilus meidingeri*	Austria (Mondsee lake)	
R. meidingeri	KR477254	*Rutilus meidingeri*	Austria (Mondsee lake)	
R. meidingeri	KR477253	*Rutilus meidingeri*	Austria (Mondsee lake)	
R. meidingeri	KR477099	*Rutilus meidingeri*	Austria (Mondsee lake)	
R. ohridanus	MG806874	*Leucos basak*	Albania (Ohrid lake)	
R. ohridanus	KJ554509	*Leucos basak*	Albania (Ohrid lake)	
R. ohridanus	KJ554414	*Leucos basak*	Albania (Ohrid lake)	
R. ohridanus	KJ554350	*Leucos basak*	Albania (Ohrid lake)	
R. panosi	MG806875	*Leucos panosi*	Greece (Trichonis lake)	
R. panosi	KJ554528	*Leucos panosi*	Greece (Trichonis lake)	
R. panosi	KJ554495	*Leucos panosi*	Greece (Trichonis lake)	
R. panosi	KJ554396	*Leucos panosi*	Greece (Trichonis lake)	
R. pigus	HM560327	*Rutilus pigus*	Italy (Adda)	
R. pigus	HM560326	*Rutilus pigus*	Italy (Adda)	
R. pigus	KJ554485	*Rutilus pigus*	Italy (Como lake)	
R. pigus	KJ554328	*Rutilus pigus*	Italy (Como lake)	
R. prespensis	KJ554482	*Leucos basak*	Albania (Prespa lake)	
R. prespensis	KJ554471	*Leucos basak*	Albania (Prespa lake)	
R. prespensis	KJ554468	*Leucos basak*	Albania (Prespa lake)	
R. prespensis	KJ554445	*Leucos basak*	Albania (Prespa lake)	
R. rubilio	KJ554475	*Sarmarutilus rubilio*	Italy (Arno)	ARN-Cas
R. rubilio	KJ554114	*Sarmarutilus rubilio*	Italy (Arno)	ARN-Cas
R. rubilio	KJ554324	*Sarmarutilus rubilio*	Italy (Arno)	ARN-Ter
R. rubilio	KJ554212	*Sarmarutilus rubilio*	Italy (Arno)	ARN-Ter
R. rubilio	KJ554352	*Sarmarutilus rubilio*	Italy (Tiber)	TIB-Ang

R. rubilio	KJ554250	*Sarmarutilus rubilio*	Italy (Tiber)	TIB-Ang
R. rubilio	KJ554361	*Sarmarutilus rubilio*	Italy (Tiber)	TIB-Mon
R. rubilio	KJ554088	*Sarmarutilus rubilio*	Italy (Tiber)	TIB-Mon
R. rubilio	KJ554380	*Sarmarutilus rubilio*	Italy (Sisto)	SIS
R. rubilio	KJ554226	*Sarmarutilus rubilio*	Italy (Sisto)	SIS
R. rubilio	KJ554435	*Sarmarutilus rubilio*	Italy (Fondi lake)	FON
R. rubilio	KJ554290	*Sarmarutilus rubilio*	Italy (Fondi lake)	FON
R. rubilio	KJ554508	*Sarmarutilus rubilio*	Italy (Volturno)	VOL
R. rubilio	KJ554364	*Sarmarutilus rubilio*	Italy (Volturno)	VOL
R. rubilio	KJ554200	*Sarmarutilus rubilio*	Italy (Volturno)	VOL
R. rubilio	KJ554118	*Sarmarutilus rubilio*	Italy (Volturno)	VOL
R. rubilio	KJ554409	*Sarmarutilus rubilio*	Italy (Crati)	CRA
R. rubilio	KJ554274	*Sarmarutilus rubilio*	Italy (Crati)	CRA
R. rubilio	KJ554129	*Sarmarutilus rubilio*	Italy (Crati)	CRA
R. rutilus	KT989765	*Rutilus rutilus*	Russia (Plescheevo lake)	
R. rutilus	HQ961042	*Rutilus rutilus*	Czech Republic (Ohre)	
R. rutilus	MW473258	*Rutilus rutilus*	Germany (Rhine)	
R. rutilus	HM392103	*Rutilus rutilus*	Germany (Danube)	
R. virgo	HM392106	*Rutilus virgo*	Germany (Danube)	
R. virgo	HM392104	*Rutilus virgo*	Germany (Vils)	
R. virgo	HM392102	*Rutilus virgo*	Germany (Danube)	
R. virgo	MG806878	*Rutilus virgo*	Croatia (Sava)	
R. ylikiensis	MG806879	*Leucos ylikiensis*	Greece (Yliki lake)	
R. ylikiensis	KJ554524	*Leucos ylikiensis*	Greece (Kifissos)	
R. ylikiensis	KJ554516	*Leucos ylikiensis*	Greece (Kifissos)	
R. ylikiensis	KJ554316	*Leucos ylikiensis*	Greece (Kifissos)	

Table S3. COI (a) and CR (b) diagnostic sites identified among the most frequent sequences of the three haplogroups found in Italian specimens. Deletions are marked with "–".

(a) Diagnostic COI (624 bp) sites

Position/ Haplotype			1	1	2	2	2	2	2	2	2	2	2	2	3	3	3	3	4	4	4	4	4	4	4	5	5	5	5	6
	5	9	4	9	1	1	5	5	5	7	7	7	8	9	0	0	3	8	0	2	3	5	6	7	9	0	2	3	5	1
	4	6	4	8	6	9	0	2	6	0	6	9	5	4	3	9	0	4	8	9	5	3	2	7	5	4	2	4	5	2
HpA01	G	T	C	G	T	G	T	G	C	C	A	T	C	A	A	G	C	G	C	T	G	A	G	A	C	C	A	A	T	C
HpB01	G	T	C	G	T	G	T	G	C	C	G	T	C	A	A	A	C	G	T	C	G	G	G	A	C	T	G	A	T	T
HpC01	A	C	T	A	C	A	C	A	T	T	A	C	T	G	G	G	T	C	T	T	A	G	A	G	A	C	G	G	C	T

(b) Diagnostic CR (930 bp) sites

Position/ Haplotype					1	3	3	3	3	4	4	4	4	4	5	5	5	6	6	6	6	7	7	7	8	8	8	8	8	8	8	8	9	9	9
		2	5	8	4	7	7	7	9	1	6	6	7	9	1	5	6	0	1	5	7	2	2	8	0	3	3	4	7	7	8	9	9	1	3
	7	5	5	6	2	1	2	6	3	7	3	5	3	4	8	5	7	8	0	3	0	4	5	3	8	5	9	4	3	7	9	4	7	6	0
HpA01	C	A	G	T	C	C	G	G	T	G	T	T	A	T	G	A	C	T	T	T	C	A	T	A	G	G	G	A	G	T	G	G	T	A	C
HpB01	G	A	A	T	C	C	G	G	A	G	C	C	A	A	G	A	T	T	T	T	T	T	T	G	A	A	G	A	G	T	A	G	T	A	C
HpC01	C	G	A	C	T	A	T	T	A	T	T	T	G	T	A	T	T	C	C	C	T	T	A	A	A	A	A	G	A	G	A	A	-	G	T

Table S4. Diagnostic fragment of nuclear marker Cyfun P in Italian specimens subset. Individuals with specific VAR sequences are highlighted in green, variable sites/indels in yellow; mtDNA lineage of each specimen is also reported. Missing data are marked with N, deletions with -.

	CyfunP GenBank Accession Number	MtDNA haplogroup	146	147	148	149	150	151	152	153	154	155	156	157	158	159	160	161	162	163	164	165	166	167	168	169	170	171	172	173	174	175	176	177	178	179	180	181	182
VAR1_46	OM966266	C	A	A	C	A	A	A	A	A	A	C	A	C	A	R	A	A	A	-	-	-	A	A	A	T	A	A	A	A	G	G	A	-	-	-	-	-	-
VAR1_49	OM966267	C	A	A	C	A	W	A	A	A	A	C	A	C	A	N	A	A	A	-	-	-	A	A	A	T	A	A	A	A	G	G	A	-	-	-	-	-	-
VAR2_02	OM966268	A	A	A	C	A	A	A	A	A	A	C	A	C	A	G	A	A	A	-	-	-	A	A	A	T	A	A	A	A	G	G	A	-	-	-	-	-	-
VAR2_09	OM966269	C	A	A	C	A	A	A	A	A	A	C	A	C	A	N	A	A	A	-	-	-	A	A	A	T	A	A	A	A	G	G	A	-	-	-	-	-	-
VAR2_11	OM966270	C	A	A	C	A	A	A	A	A	A	C	A	N	N	N	N	N	N	-	-	-	N	N	N	N	N	N	N	N	N	N	N	-	-	-	-	-	-
VAR2_25	OM966271	C	A	A	C	A	A	A	A	A	A	C	A	C	A	N	A	A	A	-	-	-	A	A	A	T	A	A	A	A	G	G	A	-	-	-	-	-	-
VAR2_32	OM966272	C	A	A	C	A	W	A	A	A	A	C	A	C	A	N	A	A	A	-	-	-	A	A	A	T	A	A	A	A	G	G	A	-	-	-	-	-	-
VAR1_33	OM966273	A	T	A	A	A	A	A	A	A	T	A	A	A	A	N	T	A	A	A	A	T	A	A	A	T	A	A	A	N	N	N	N	N	N	N	N	N	N
VAR1_43	OM966274	A	T	A	A	A	W	A	A	A	T	A	A	A	A	A	T	A	A	A	A	N	N	N	N	N	N	N	N	N	N	N	N	N	N	N	N	N	N
VAR2_04	OM966275	C	T	A	A	A	A	A	A	A	T	A	A	A	A	A	T	A	A	A	A	T	A	A	A	T	A	A	N	N	N	N	N	N	N	N	N	N	N
VAR2_05	OM966276	C	T	A	A	A	A	A	A	A	T	A	A	A	A	A	T	A	A	A	A	T	A	A	A	T	A	A	A	A	G	G	A	A	G	T	T	T	C
VAR2_06	OM966277	C	T	A	A	A	A	A	A	A	T	A	A	A	A	A	T	A	A	A	A	T	A	A	A	T	A	A	A	A	G	G	A	A	G	T	T	T	C
VAR2_28	OM966278	A	T	A	A	A	A	A	A	A	T	A	A	A	A	A	T	A	A	A	A	T	A	A	A	T	A	A	A	A	G	G	A	A	G	T	T	T	C
TIB1_26	OM966279	A	T	A	A	A	T	A	A	A	T	A	A	A	A	A	T	A	A	A	A	T	A	A	A	N	N	N	N	N	N	N	N	N	N	N	N	N	N
SCR_01	OM966280	A	T	A	A	A	A	A	A	A	T	A	A	A	A	A	T	A	A	A	A	T	A	A	A	T	A	A	A	A	G	G	A	A	G	T	T	T	C
SCR_02	OM966281	A	T	A	A	A	A	A	A	A	T	A	A	A	A	A	T	A	A	A	A	T	A	A	A	T	A	A	A	A	G	G	A	A	G	T	T	T	C

Table S5. Inter- (below the diagonal) and intra-haplogroup (diagonal, in grey) genetic mean distances (CR); pairwise ΦST between CR haplogroups (above the diagonal).

	A	B	C
A	0.0009 (±0.0005)	0.94***	0.97***
B	0.0151 (±0.0040)	0.0013 (±0.0005)	0.96***
C	0.0325 (±0.0062)	0.0291 (±0.0058)	0.0005 (±0.0005)

Table S6. Population Φ_{ST} (CR) considering all the populations as they are (a) and splitting individuals by haplogroup for each one (b). Significance thresholds: * = P < 0.05; ** = P < 0.01; *** = P < 0.001.

(a)

		Haplogroup A/C		Haplogroup A						Haplogroup A/B		Haplogroup B	
		VAR1	VAR2	TRO	FOR	ARR	TIB1	TIB2	GAR	SCR	NOC	SET	SPU
HpA/C	VAR1	-											
	VAR2	-0.05	-										
HpA	TRO	0.29	0.3	-									
	FOR	0.28	0.33	0.05	-								
	ARR	0.31***	0.35	0.68***	0.64***	-							
	TIB1	0.38***	0.43***	0.13	0.13	0.45***	-						
	TIB2	0.06	0.16	0.20	0.19	0.47	0.13	-					
	GAR	0.33	0.38	0.07	0.08	0.61***	0.15***	0.01	-				
HpA/B	SCR	0.32***	0.38**	0.18	0.17	0.36***	0.21***	0.01	0.11	-			
	NOC	0.09	0.16	0.42***	0.38***	0.31***	0.41***	0.01	0.36***	0.12	-		
HpB	SET	0.65***	0.58***	0.98***	0.97***	0.96***	0.95***	0.96***	0.97***	0.90***	0.87***	-	
	SPU	0.51***	0.47***	0.98***	0.96***	0.95***	0.95***	0.95	0.97***	0.88***	0.79***	0.12	-

(b)

		Haplogroup C		Haplogroup A										Haplogroup B			
		VAR1_C	VAR2_C	VAR1_A	VAR2_A	TRO	FOR	ARR	TIB1	TIB2	SCR_A	GAR	NOC_A	SET	SPU	SCR_B	NOC_B
HpC	VAR1_C	-															
	VAR2_C	-0.07	-														
HpA	VAR1_A	0.99	0.99***	-													
	VAR2_A	0.99	0.99***	-0.03	-												
	TRO	0.99***	0.99***	-0.04	0.01	-											
	FOR	0.99	0.99***	0.02	0.04	0.05	-										
	ARR	0.97***	0.98***	0.63	0.63***	0.68***	0.64***	-									
	TIB1	0.97***	0.98***	0.09	0.10	0.13	0.13	0.45***	-								
	TIB2	0.97	0.98	0.22	0.18	0.20	0.19	0.47	0.13	-							
	SCR_A	0.97***	0.97***	0.23	0.19	0.28**	0.27**	0.49***	0.28***	0.09	-						
	GAR	0.99***	0.99**	0.03	0.03	0.07	0.08*	0.61***	0.15***	0.01	0.17	-					
	NOC_A	0.99	0.99***	1.00***	0.89	0.92***	0.84***	0.64***	0.60***	0.55	0.35	0.67	-				
HpB	SET	0.98***	0.98***	0.97***	0.97***	0.98***	0.97***	0.96***	0.95***	0.96***	0.94***	0.97***	0.97***	-			
	SPU	0.98	0.98***	0.98***	0.98***	0.98***	0.98***	0.95***	0.95***	0.95	0.93***	0.97***	0.98	0.12	-		
	SCR_B	0.98	0.98	1.00	0.99	0.99	0.98	0.94	0.95	0.94	0.93	0.98	1.00	0.89	0.87	-	
	NOC_B	0.98	0.98	1.00	0.99	0.99	0.98	0.94	0.95	0.94	0.93	0.98	1.00	0.92	0.91	1.00	-

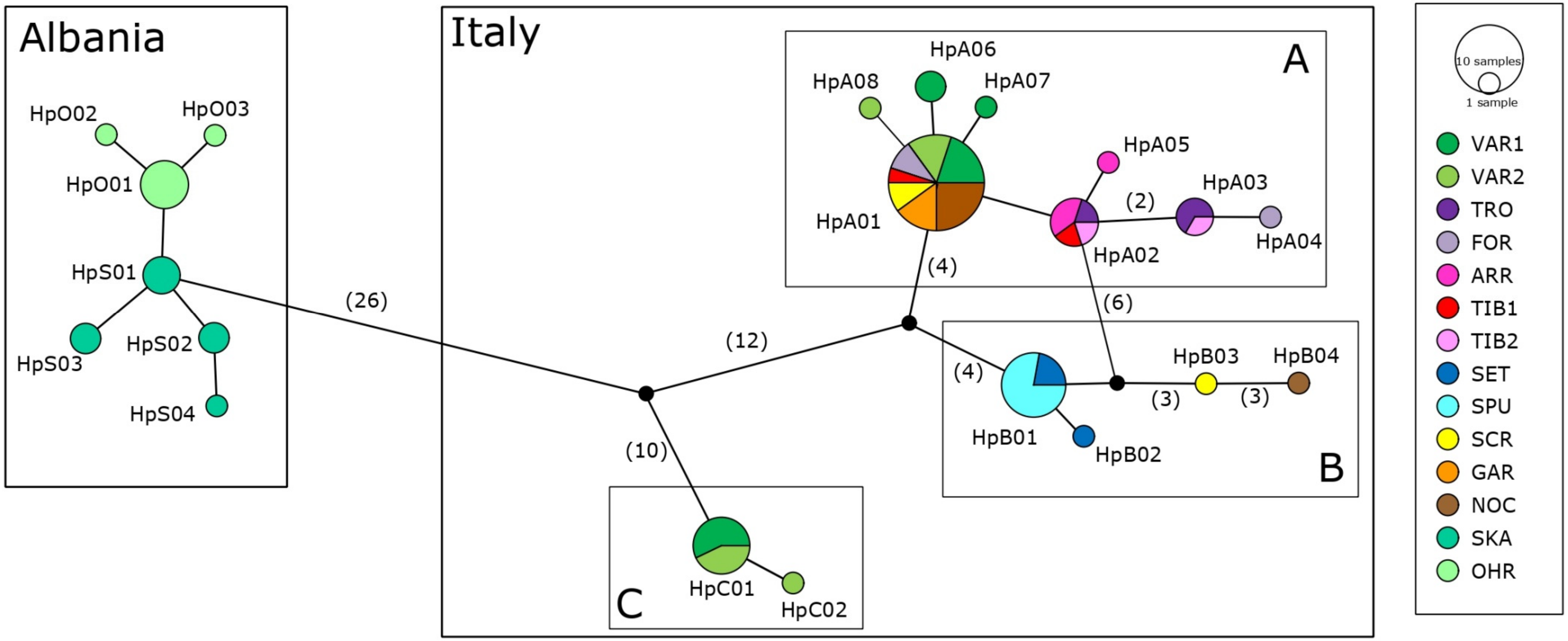

Figure S1. Haplotype network based on COI sequences obtained in this study. Each circle corresponds to one haplotype and its dimension is proportional to the haplotype frequency. The number of nucleotide substitutions between haplotypes is indicated in parenthesis. Population abbreviations refer to Table 1. Letters (A, B, C) and boxes refer to *S. rubilio* haplogroups as indicated in Figure 2.

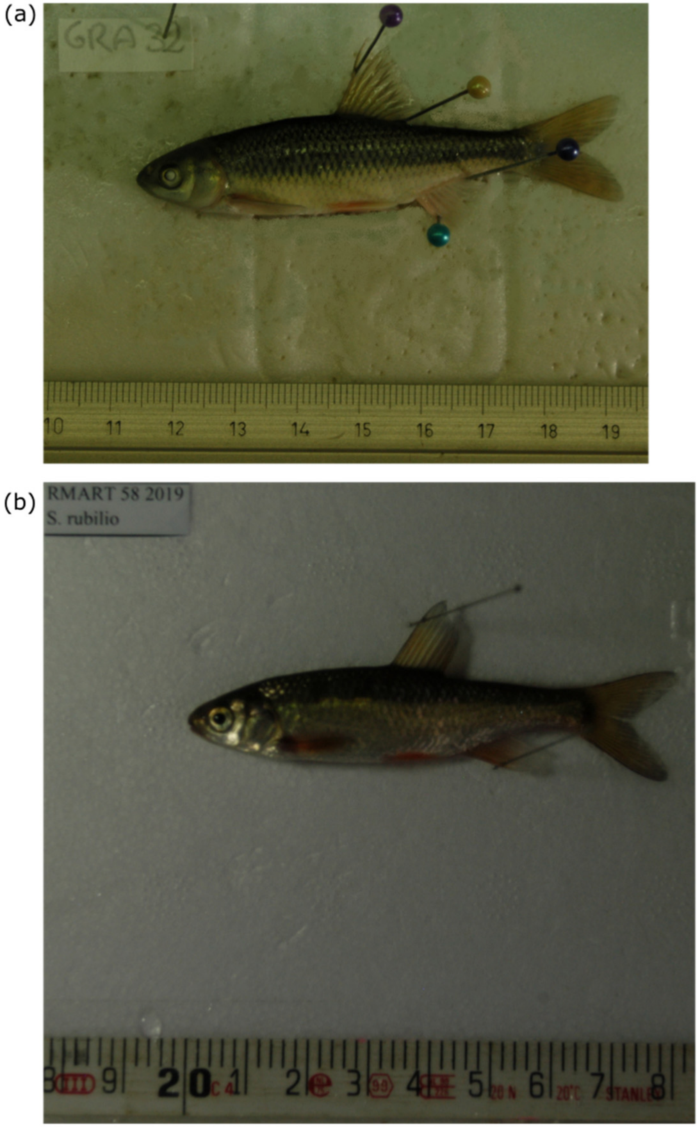

Figure S2. Specimens with mtDNA and Cyfun P belonging to lineage C (a) and lineage A (b).

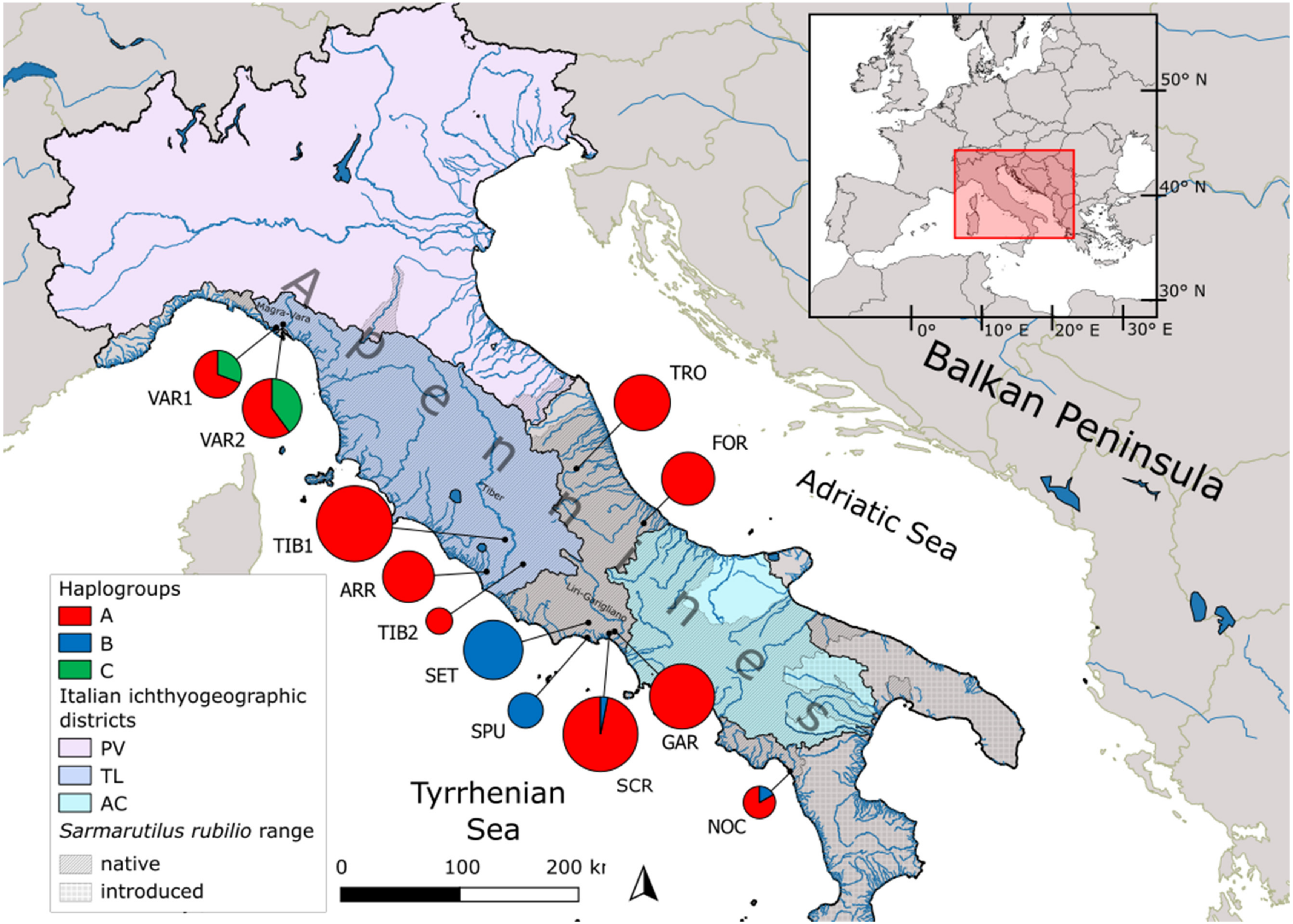

Figure S3. Spatial haplogroups distribution in *S. rubilio*, based on 208 Italian CR sequences.

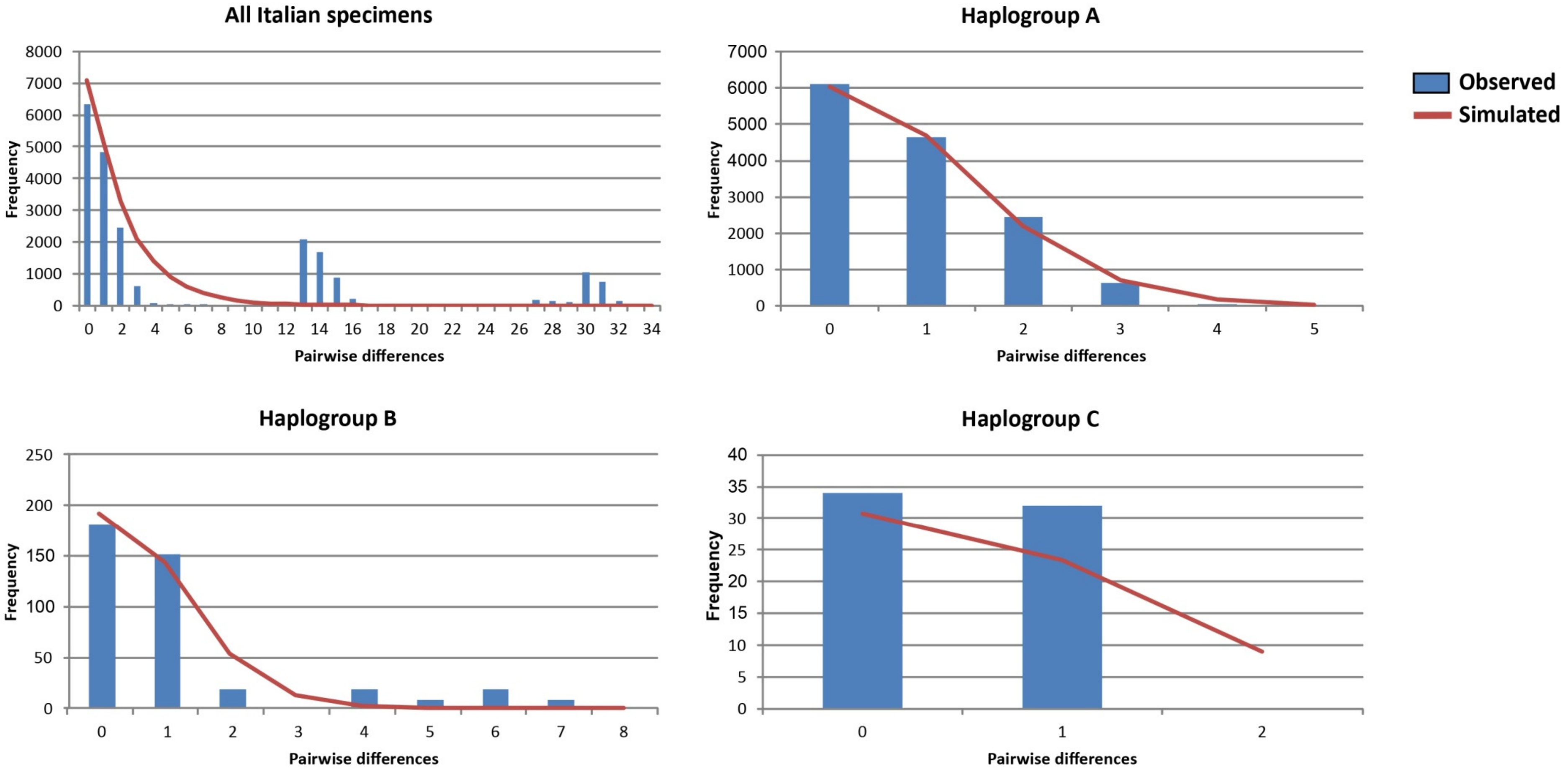

Figure S4. Observed mismatch distributions (bars) and expected mismatch distributions under the sudden expansion model (solid line) of CR sequences.
